# Supplementary material for: Domain memory effect in the organic ferroics
Source: Nat Commun. 2022 May 2;13:2379. doi: 10.1038/s41467-022-30085-1 (PMC9061795; doi:10.1038/s41467-022-30085-1)
Supplement: Supplementary file 1 — Supplementary Information [file 41467_2022_30085_MOESM1_ESM.pdf]

Supplementary Information

Domain Memory Effect in the Organic Ferroics

Zhong-Xia Wang[1,2]*, Xiao-Gang Chen[2], Xian-Jiang Song[2], Yu-Ling Zeng[2], Peng-Fei Li[2], Yuan-Yuan Tang[2], Wei-Qiang Liao[2], and Ren-Gen Xiong[2]*

[1]College of Chemistry and Chemical Engineering, Gannan Normal University, Ganzhou 341000, People's Republic of China.
[2]Ordered Matter Science Research Center, Nanchang University, Nanchang 330031, People's Republic of China.

*Email: zhongxiawang@ncu.edu.cn; xiongrg@ncu.edu.cn

Measurement Methods

Single-crystal X-ray crystallography. Crystallographic data were collected using a Rigaku Saturn 924 diffractometer equipped with a temperature control device, by using Cu Kα (λ = 1.54187 Å) radiation. Data processing including empirical absorption correction, cell refinement, and data reduction was performed using the Crystal Clear software package. The data collection and structure refinement of these crystals are summarized in Tables S1-S3. Powder X-ray diffraction (PXRD) data were measured using a Rigaku D/MAX 2000 PC X-ray diffraction system with Cu K*α* radiation in the 2θ range of 5°–40° with a step size of 0.02° and a scan rate of 10°/min.

DSC and SHG measurements. Differential scanning calorimetry (DSC) measurements were performed on a PerkinElmer Diamond DSC under nitrogen atmosphere in aluminum crucibles with a heating or cooling rate of 10 K/min. For second harmonic generation (SHG) experiments, an unexpanded laser beam with low divergence (pulsed Nd:YAG at a wavelength of 1064 nm, 5 ns pulse duration, 1.6 MW peak power, 10 Hz repetition rate) was used. The instrument model is Ins 1210058, INSTEC Instruments, while the laser is Vibrant 355 II, OPOTEK. The numerical values of the nonlinear optical coefficients for SHG have been determined by comparison with a KDP reference.

UV-vis diffuse-reflectance spectra, CD spectra, VCD and IR measurements. The thin film and powder samples were used for UV-vis measurements and the potassium bromide (KBr) tablets method was used for CD, VCD and IR measurements. The UV-vis and CD spectra are recorded with SHIMADZU UV-3600Plus equipped with ISR-603 integrating sphere and JASCO J-1700, respectively. The spectra are recorded with Bruker INVENIO and PMA-50 module. $BaSO_4$ was used as a 100% reflectance reference. The thin film was detected in transmission mode, and the powder sample was detected in diffuse reflection mode. The photoreaction time for UV-vis measurement is 40s under 365 nm UV light and 10s under 488 nm visible light. For CD, VCD and IR spectroscopy tests, the photoreaction time is up to 60s (Fig. S24).

Calculation condition. The HOMO, LUMO, VCD and IR are calculated at b3lyp/6-31G(d) level with Gaussian 09 software. We constructed enol, *cis*-keto and *trans*-keto configurations based on the experimentally measured single-crystal X-ray diffraction structure.

***P*−*E* hysteresis loop measurements.** A thin-film crystal capacitor was fabricated for the *P*–*E* hysteresis loop measurement. For the film, GaIn eutectic was used as the top electrode. The ferroelectric hysteresis measurements were conducted with this capacitor architecture (GaIn/sample film/ITO) under the double wave method.

PFM characterization. The PFM measurement was carried out on a commercial piezoresponse force microscope (Oxford instrument, Cypher ES) with a high-voltage package, in-situ heating stage, and custom-designed light sources. Conductive Pt/Ir-coated silicon probes (EFM, Nanoworld) were used for domain imaging and polarization switching studies, with a nominal spring constant of ~2.8 nN/nm

and a free-air resonance frequency of ~75 kHz. Since the amplitude of the low-frequency vertical PFM was within the noise level of the quadrant photodetector of the AFM, we performed the PFM experiments at contact resonance. The typical drive frequency was in the range of 300 to 420 kHz for vertical PFM images and 600 to 720 kHz for lateral PFM images, depending on the contact resonant frequency.

UV irradiation. UV 365 nm lamp (3000 mW/cm^2) and visible 488 nm lamp (60 mW/cm^2) were used for irradiation.

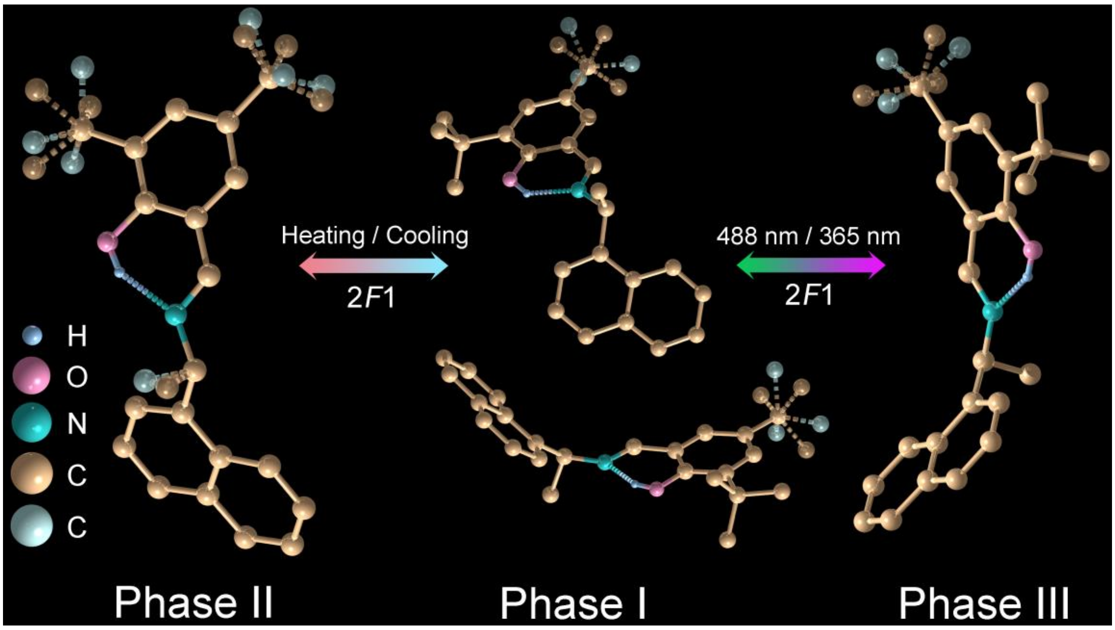

Figure S1. Asymmetric units of SA-NPh-(*S*) in Phase I, II and III. The dotted lines between N and H atoms indicate O−H···N hydrogen bonding. Some H atoms were omitted for clarity. The disordered *tert*-butyl and methyl substituents are distinguished by tan and light turquoise colors.

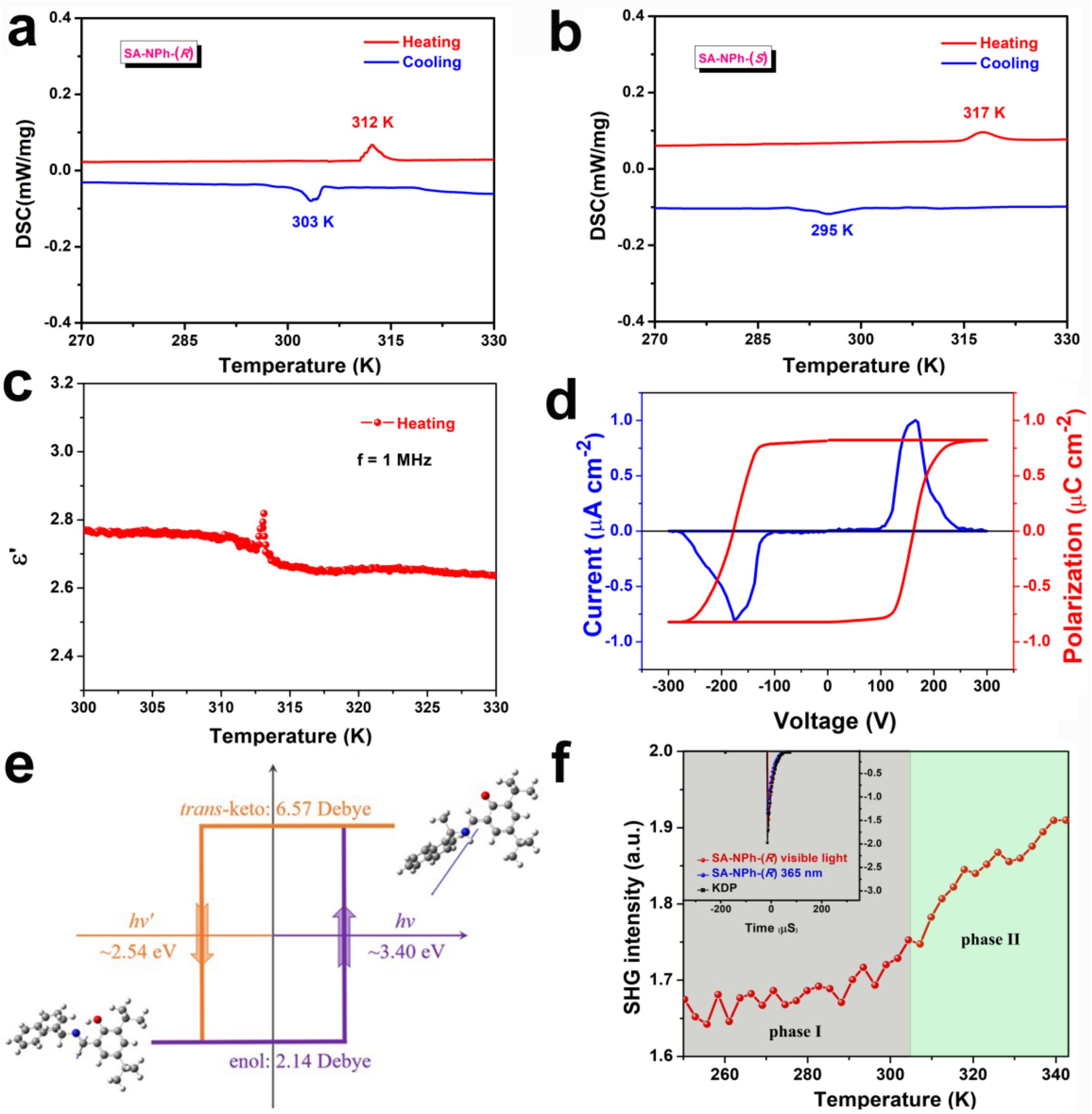

Figure S2. Phase transformation and ferroelectric properties. a,b) DSC curves of SA-NPh-(*R*) and SA-NPh-(*S*). c) The real part (ε') of the dielectric permittivity of SA-NPh-(*R*) measured on the single crystal along the crystallographic *c*-axis. d) Current density-voltage (J−V) curve and the corresponding polarization–voltage (P–V) hysteresis loop (red line) of SA-NPh-(*R*) at room temperature. The current peaks (blue line) are ascribed to the ferroelectric switching. e) Schematic diagram of molecular conformation transformation of SA-NPh-(*S*). f) Temperature-dependent SHG response for SA-NPh-(*R*). Inset: Comparison of SHG intensity in Phase I and Phase III.

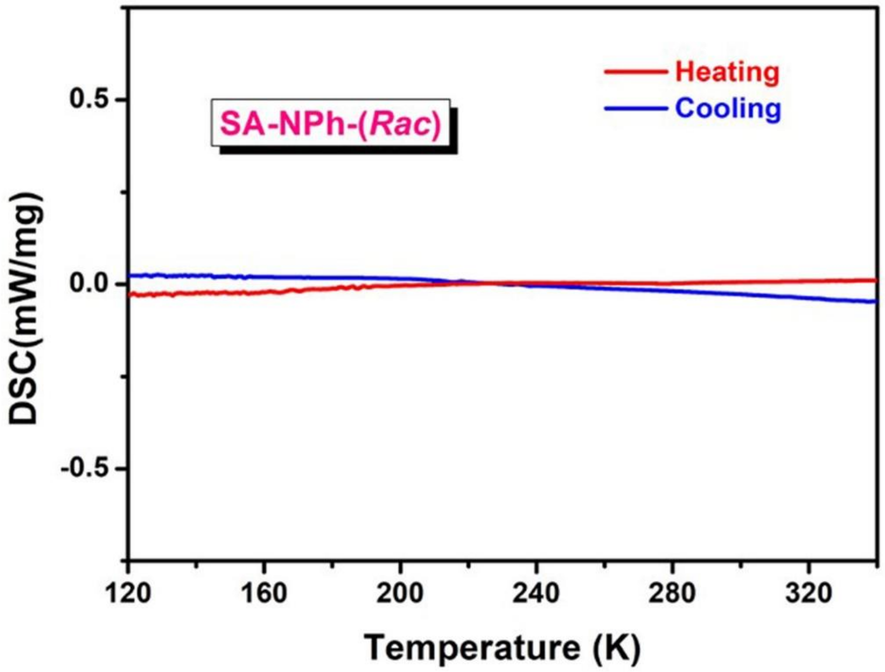

Figure S3. DSC curves of SA-NPh-(*Rac*).

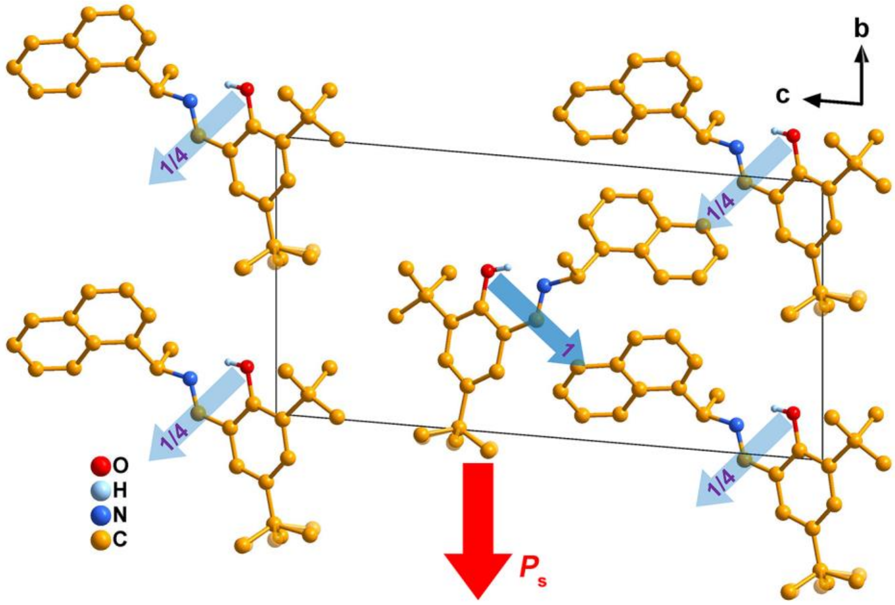

Figure S4. Schematic diagram of the molecular dipole (blue arrows) direction and the total polarization (red arrow) direction of the unit cell in Phase I with space group *P*1.

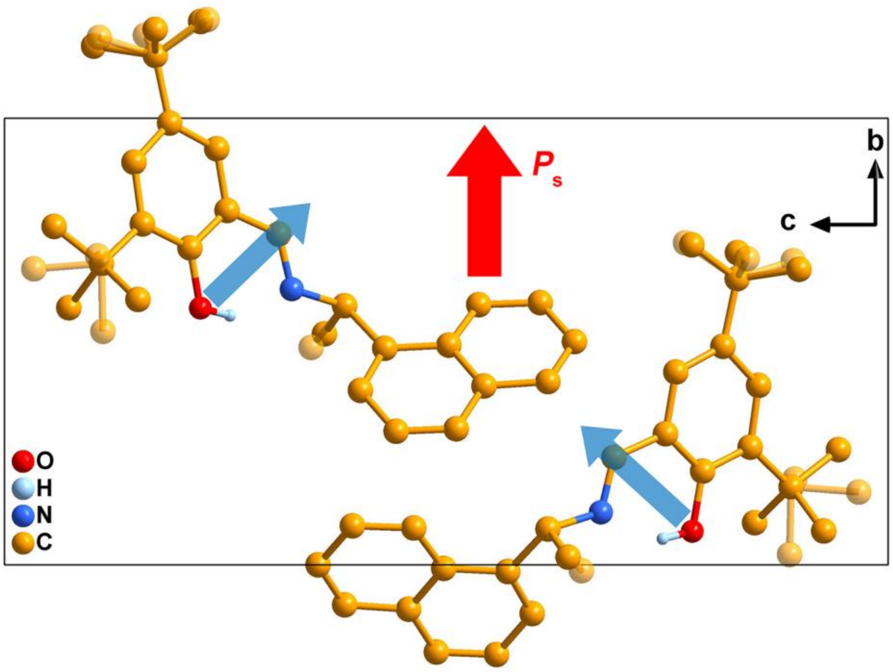

Figure S5. Schematic diagram of the molecular dipole (blue arrows) direction and the total polarization (red arrow) direction of the unit cell in Phase II with space group $P2_1$.

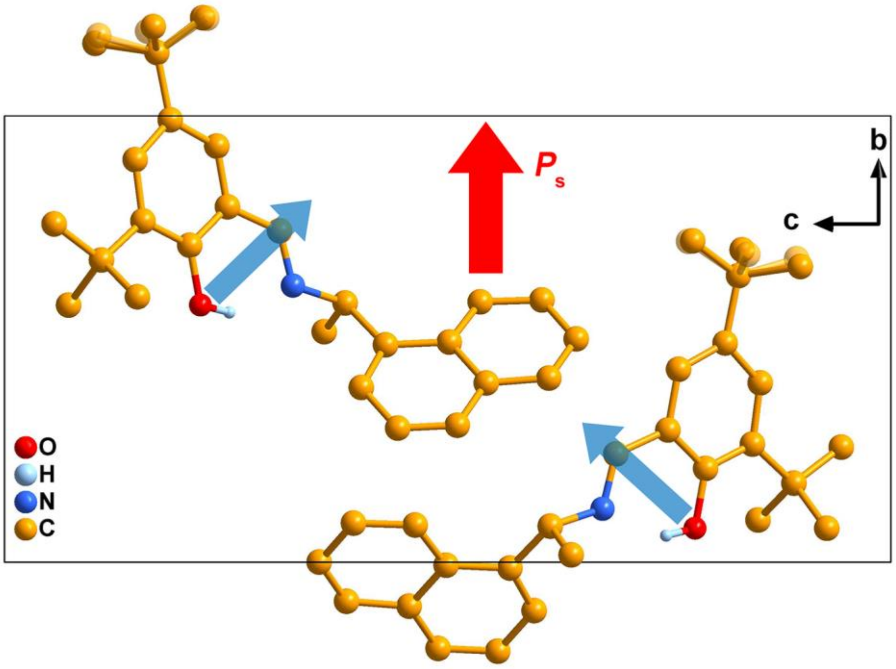

Figure S6. Schematic diagram of the molecular dipole (blue arrows) direction and the total polarization (red arrow) direction of the unit cell in Phase III with space group $P2_1$.

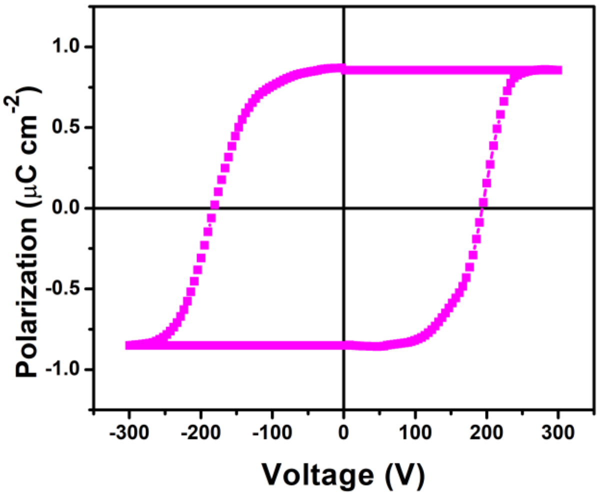

Figure S7. Polarization–voltage (*P*–*V*) hysteresis loop of SA-NPh-(*R*) at 323 K in Phase II.

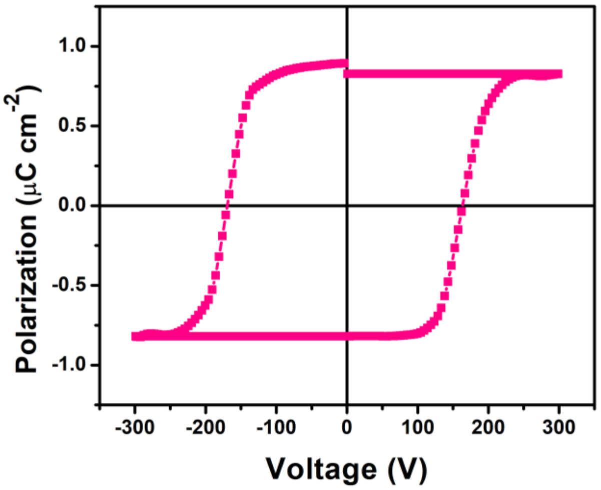

Figure S8. Polarization–voltage (*P*–*V*) hysteresis loop of SA-NPh-(*R*) by 365 nm UV light in Phase III.

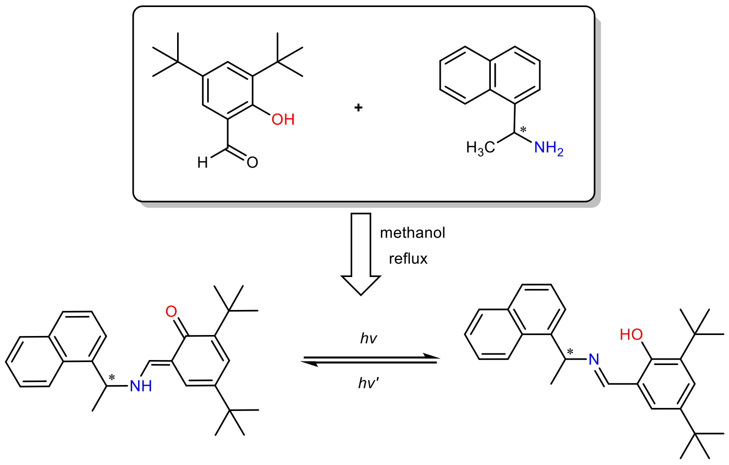

Figure S9. General procedure for the preparation of SA-NPh-(*R*), SA-NPh-(*S*) and SA-NPh-(*Rac*). The symbol * represents the *R*, *S* and *Rac* configurations.

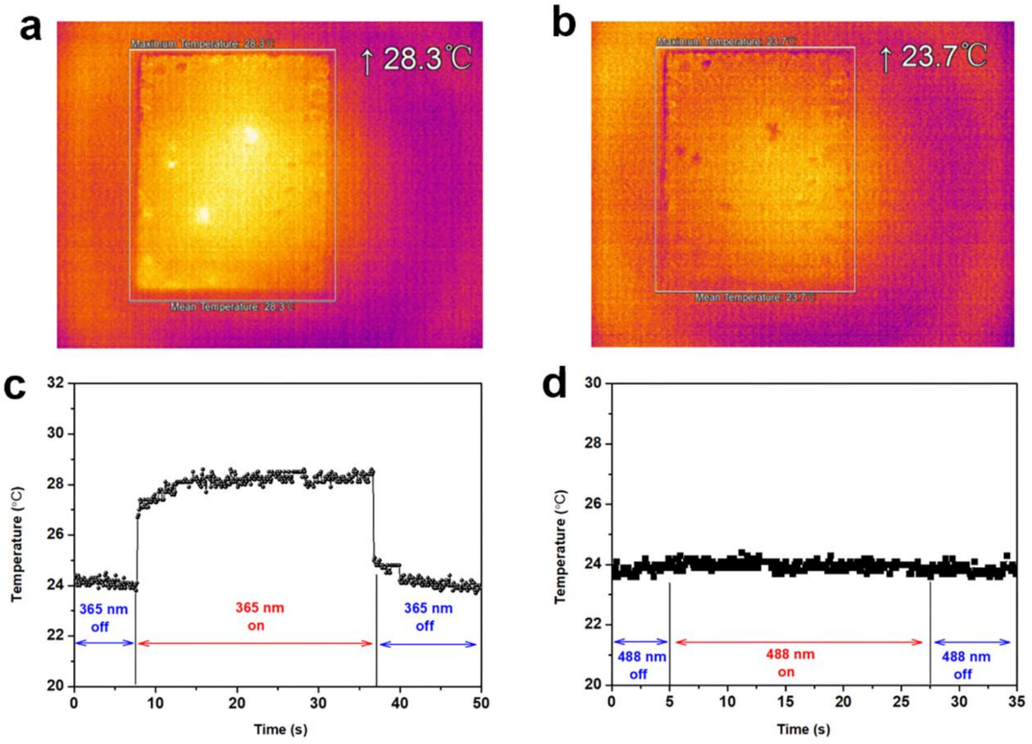

Figure S10. Infrared thermal imaging analyses of SA-NPh-(*S*). a) sample in enol form irradiated under 365 nm UV light. b) sample in *trans*-keto form irradiated under 488 nm visible light. c) temperature variation with the 365 nm UV light on and off. d) temperature variation with the 488 nm visible light on and off.

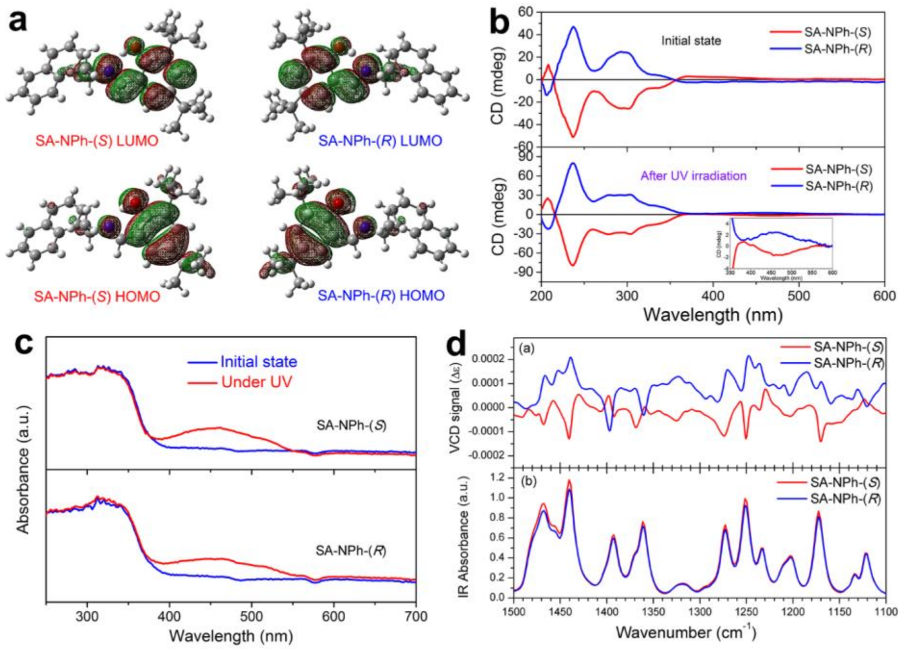

Figure S11. Electronic structure and spectral characterization. a) HOMO and LUMO of SA-NPh-(*S*) and SA-NPh-(*R*). b) Experimental electronic CD spectra of SA-NPh-(*S*) and SA-NPh-(*R*) at ambient condition and under UV irradiation of 365 nm. c) Experimental UV-vis absorption spectra of SA-NPh-(*S*) and SA-NPh-(*R*) at ambient condition and under UV irradiation of 365 nm. The inset shows the zoomed views of experimental UV-vis absorption spectra under UV irradiation of 365 nm. d) VCD and IR spectra of SA-NPh-(*S*) and SA-NPh-(*R*) at 293 K.

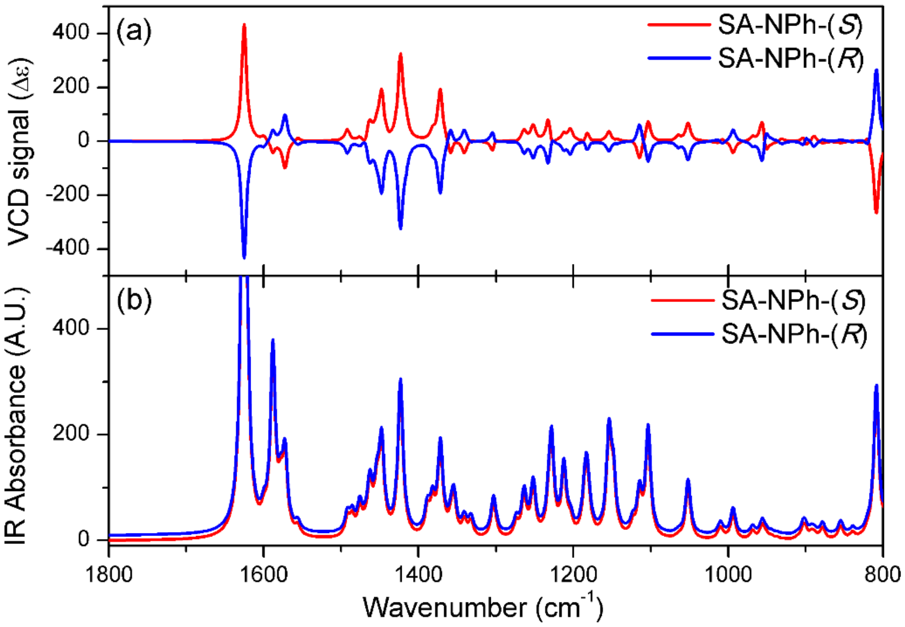

Figure S12. Calculated VCD and IR spectra of SA-NPh-(*S*) and SA-NPh-(*R*) at b3lyp/6-31G(d) level.

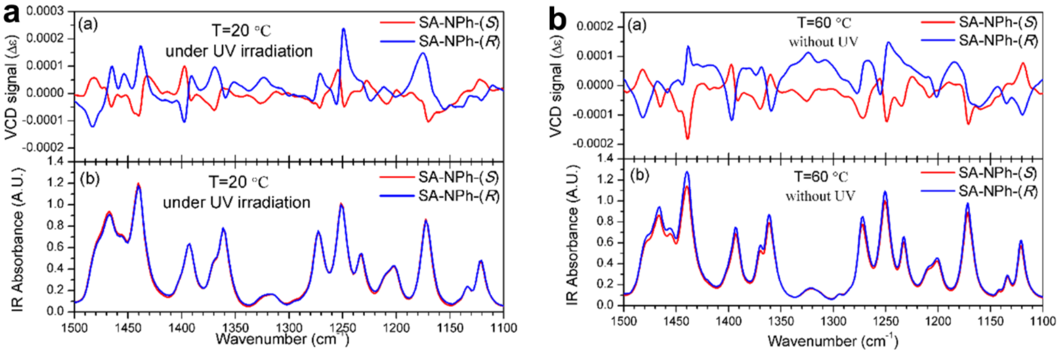

Figure S13. VCD and IR spectra. a) SA-NPh-(*S*) and SA-NPh-(*R*) at 20°C under UV radiation. b) SA-NPh-(*S*) and SA-NPh-(*R*) at 60°C without UV radiation.

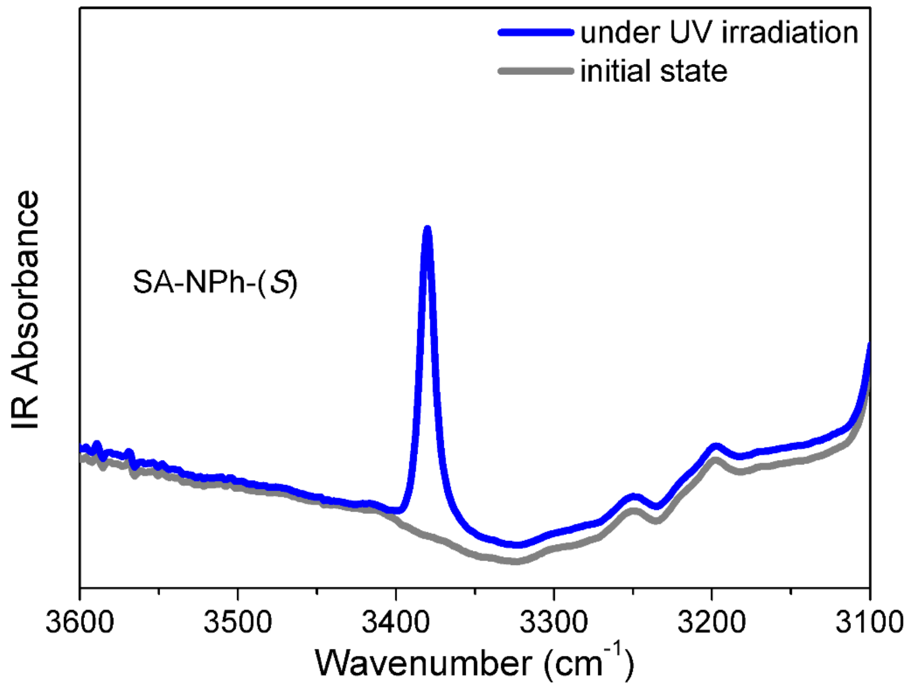

Figure S14. Experimental IR absorption spectra of SA-NPh-(*S*) under ambient condition and UV radiation (365 nm).

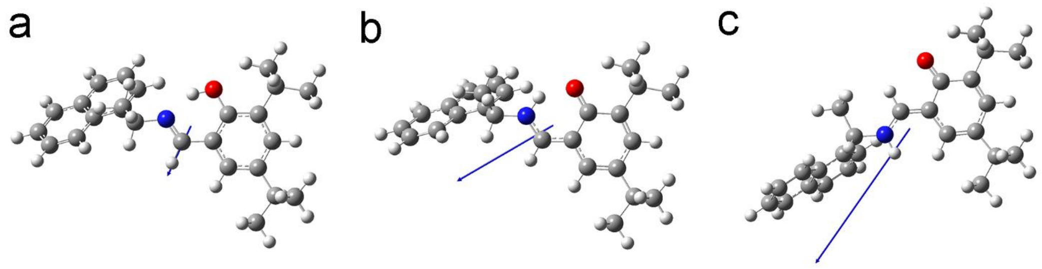

Figure S15. Molecular configure. a) SA-NPh-(*S*) in enol form, the blue arrow indicates the dipole with 2.1373 Debye. b) SA-NPh-(*S*) in *cis*-keto form. The blue arrow indicates the dipole with 4.2812 Debye. c) SA-NPh-(*S*) in *trans*-keto form. The blue arrow indicates the dipole with 6.5729 Debye.

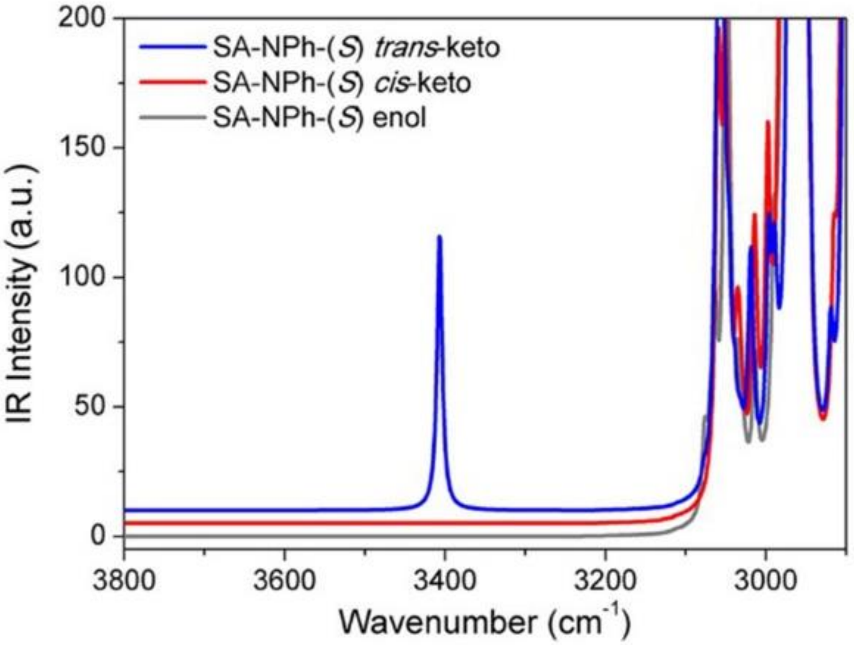

Figure S16. Calculated IR absorption spectra of SA-NPh-(*S*) with enol, *cis*-keto and *trans*-keto forms, respectively.

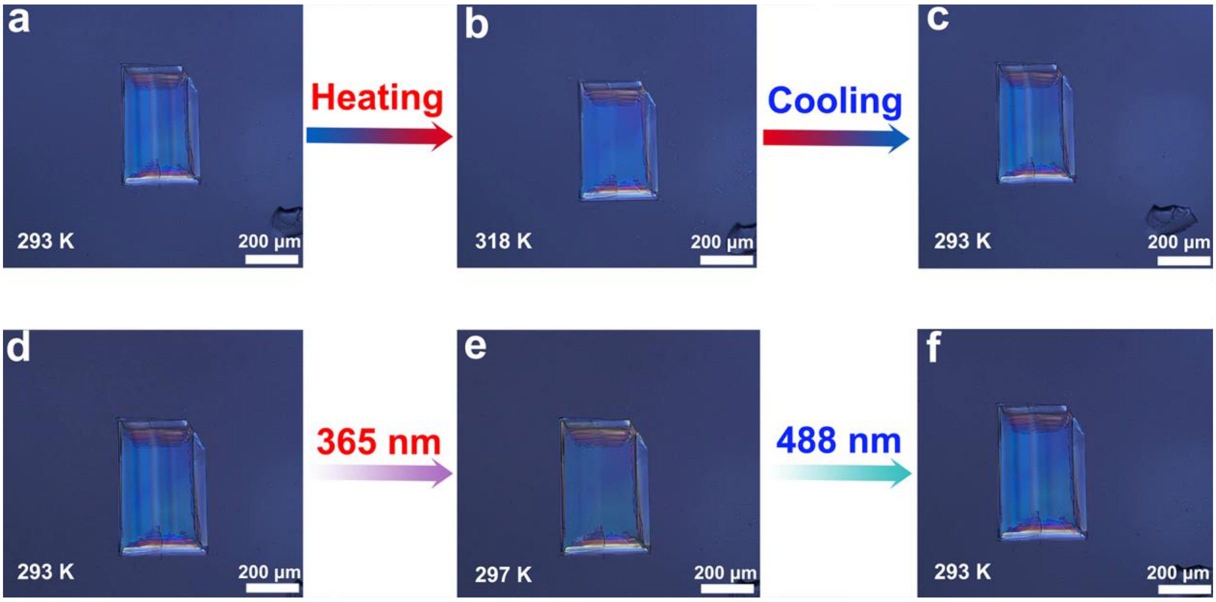

Figure S17. The evolution of ferroelastic domains under the variation of temperature and light for SA-NPh-(*R*), the scale bar is 200 μm.

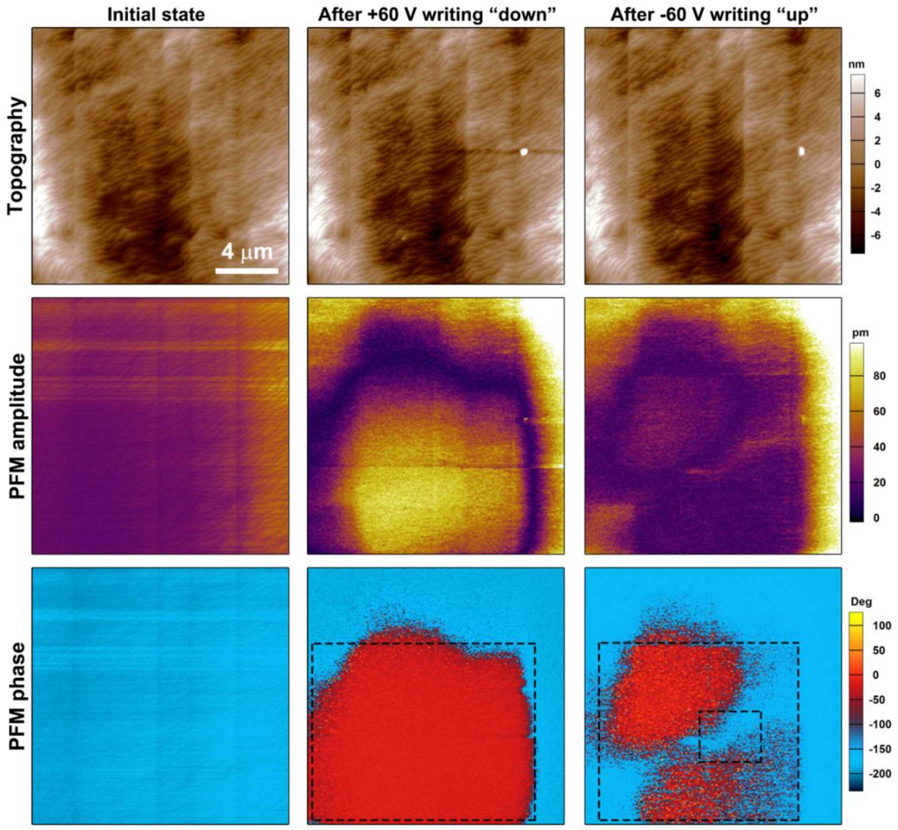

Figure S18. Domain switching for SA-NPh-(*R*) in Phase I. The topography, PFM amplitude, PFM phase for the initial state, after +60 V, and -60 V bias writing, respectively. The red and blue regions represent the two different states of ferroelectric domains.

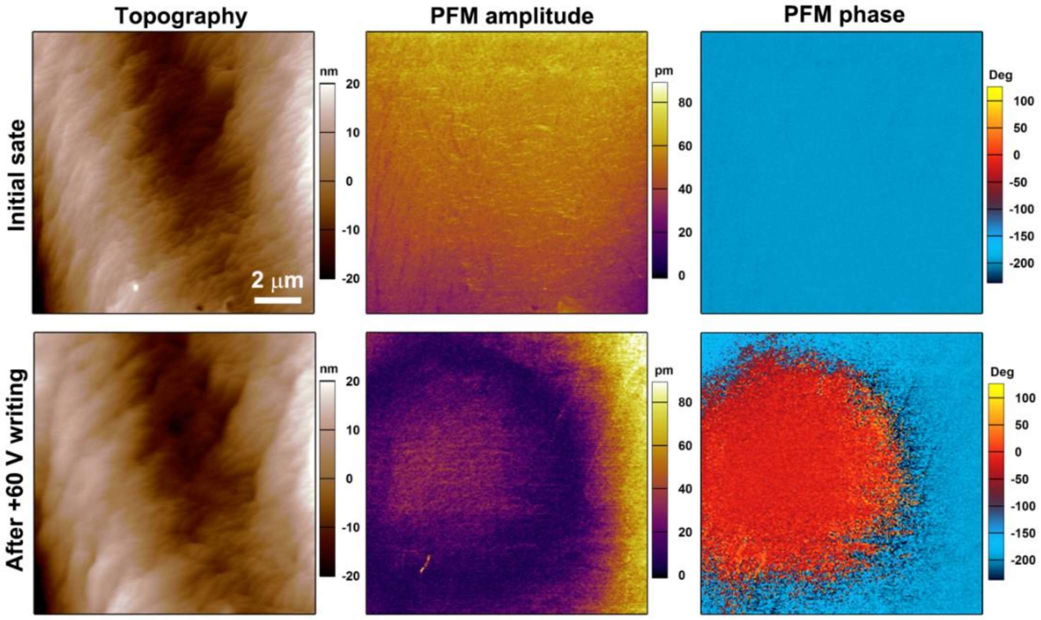

Figure S19. Domain switching for SA-NPh-(*R*) in Phase II. The topography, PFM amplitude, PFM phase for the initial state, after +60 V bias writing, respectively. The red and blue regions represent the two different states of ferroelectric domains.

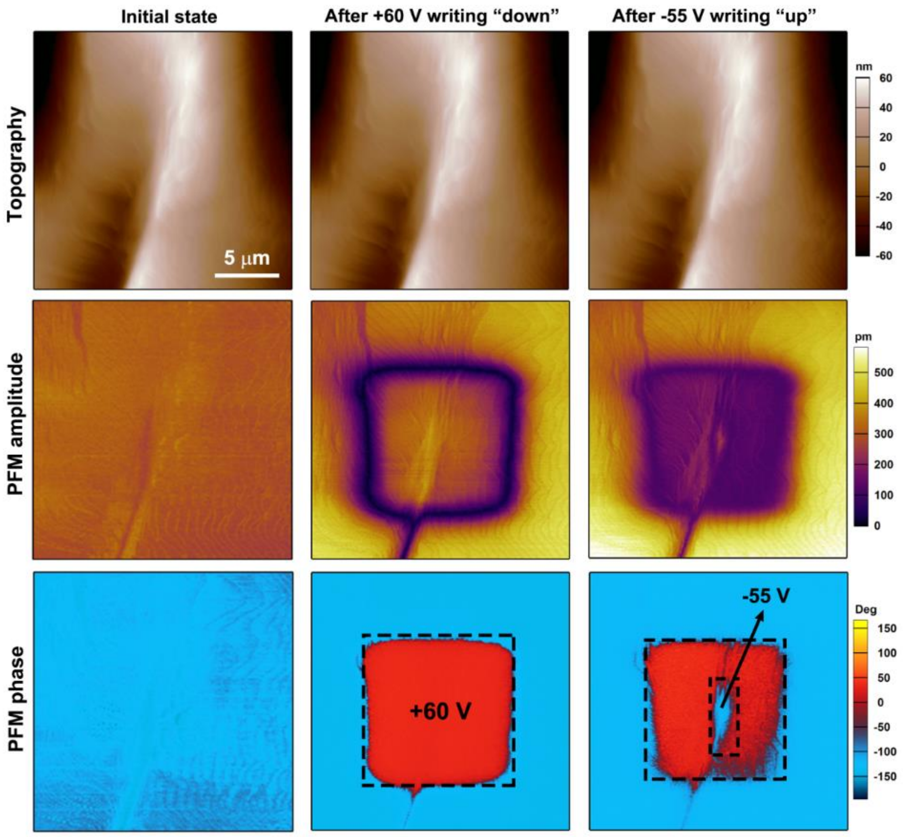

Figure S20. Domain switching for SA-NPh-(*S*) in Phase I. The topography, PFM amplitude, PFM phase for the initial state, after +60 V, and -55 V bias writing, respectively. The red and blue regions represent the two different states of ferroelectric domains.

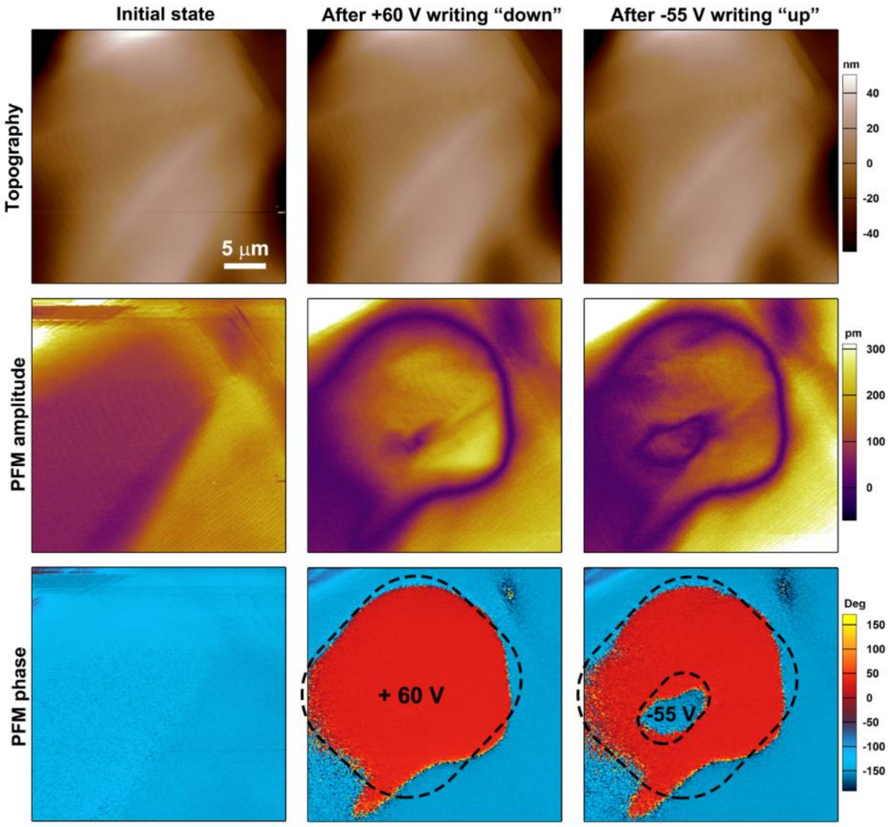

Figure S21. Domain switching for SA-NPh-(*S*) in Phase II. The topography, PFM amplitude, PFM phase for the initial state, after +60 V, and -55 V bias writing, respectively. The red and blue regions represent the two different states of ferroelectric domains.

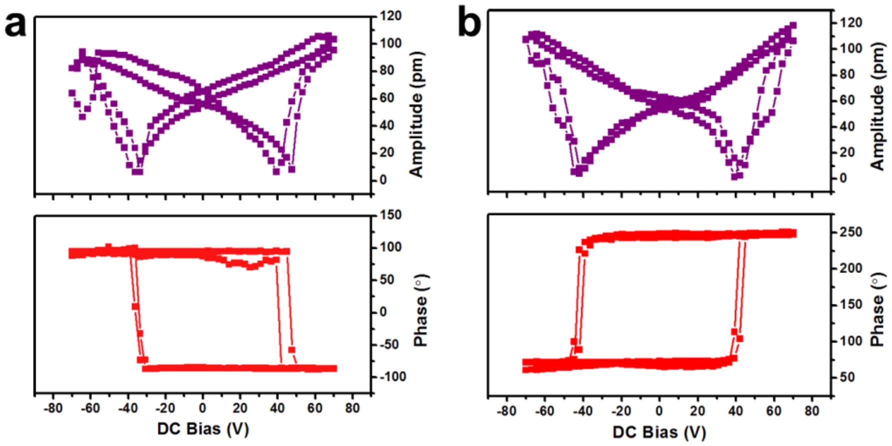

Figure S22. Amplitude and phase switching spectroscopy loops for SA-NPh-(*S*) thin film a) after 365 nm UV light irradiation and b) after 488 nm visible light irradiation.

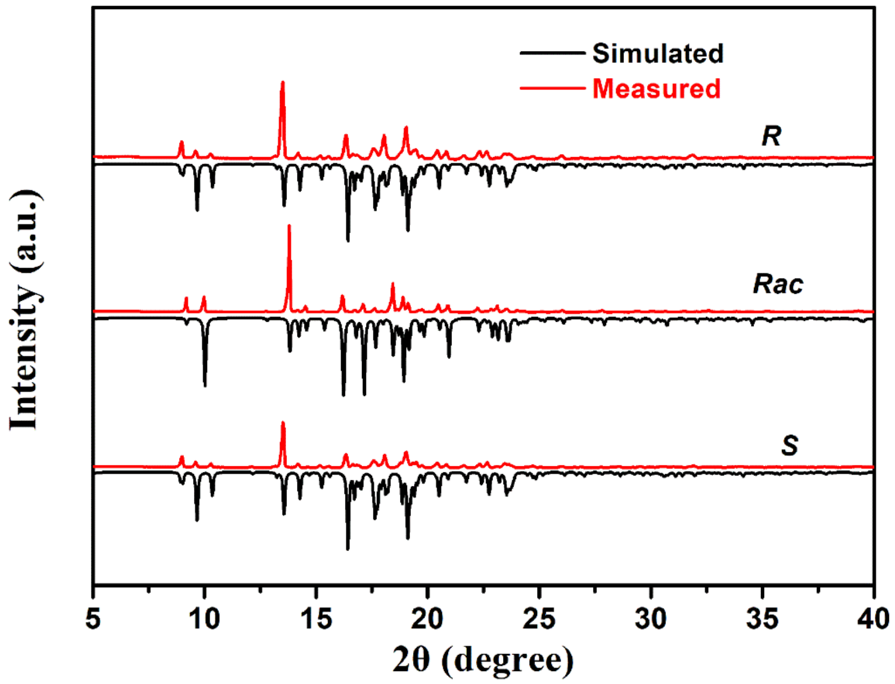

Figure S23. The measured PXRD patterns of SA-NPh-(*R*), SA-NPh-(*Rac*) and SA-NPh-(*S*) at 293 K match well with the simulated ones from crystal data at 273 K, verifying the good phase purity.

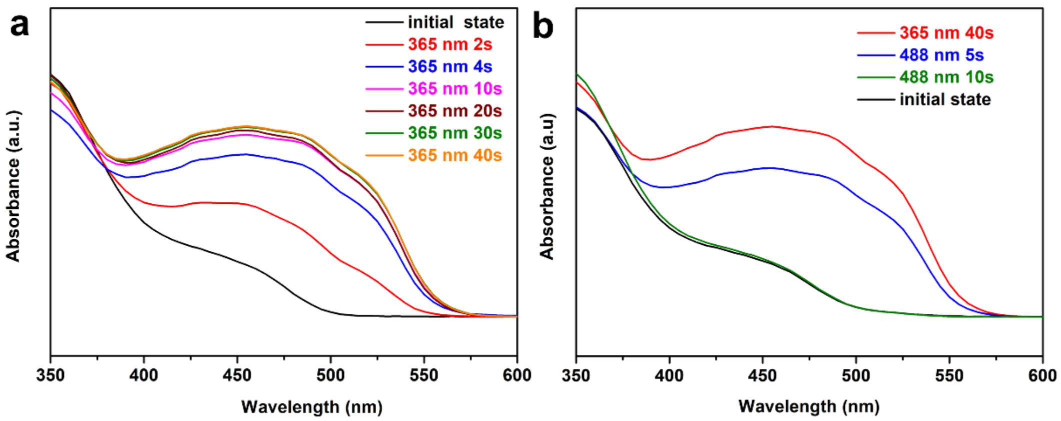

Figure S24. The time-related solid-state UV-vis absorption spectra of SA-NPh-(*R*) polycrystalline powder sample. a) 365 nm light irradiation. b) photosaturation sample under 488 nm light irradiation.

Table S1. Crystal data and structure refinement for SA-NPh-(*Rac*), SA-NPh-(*R*) and SA-NPh-(*S*) at 273 K.

	SA-NPh-(*Rac*)	SA-NPh-(*R*)	SA-NPh-(*S*)
Empirical formula	$C_{27}H_{33}NO$	$C_{27}H_{33}NO$	$C_{27}H_{33}NO$
Formula weight	387.54	387.54	387.54
Crystal system	Monoclinic	Triclinic	Triclinic
Space group	$P2_1/n$	$P1$	$P1$
a (Å)	6.24350(10)	6.2112(2)	6.2128(3)
b (Å)	9.92490(10)	9.9212(2)	9.9271(3)
c (Å)	38.4754(3)	19.6649(3)	19.6713(7)
α (°)	90	85.0730(10)	85.091(3)
β (°)	93.9070(10)	86.269(2)	86.286(4)
γ (°)	90	88.594(2)	88.577(3)
Volume (Å^3)	2378.63(5)	1204.55(5)	1221.81(6)
Z	4	2	2
GOF	1.003	1.004	1.003
R_1 [$I > 2\sigma(I)$]	0.0520	0.0641	0.0791
wR_2 [$I > 2\sigma(I)$]	0.1414	0.1894	0.2169

Table S2. Crystal data and structure refinement for SA-NPh-(*R*) and SA-NPh-(*S*) at 323 K.

	SA-NPh-(*R*)	SA-NPh-(*S*)
Empirical formula	$C_{27}H_{33}NO$	$C_{27}H_{33}NO$
Formula weight	387.54	387.54
Crystal system	Monoclinic	Monoclinic
Space group	$P2_1$	$P2_1$
a (Å)	6.2667(2)	6.2699(4)
b (Å)	9.9225(2)	9.9236(7)
c (Å)	19.7687(5)	19.7742(13)
α (°)	90	90
β (°)	96.306(3)	96.231(7)
γ (°)	90	90
Volume (Å^3)	1221.81(6)	1223.08(14)
Z	2	2
GOF	1.004	1.004
R_1 [$I > 2\sigma(I)$]	0.0698	0.0737
wR_2 [$I > 2\sigma(I)$]	0.2160	0.1996

Table S3. Crystal data and structure refinement for SA-NPh-(*Rac*), SA-NPh-(*R*) and SA-NPh-(*S*) upon UV light of 365 nm.

	SA-NPh-(*Rac*)	SA-NPh-(*R*)	SA-NPh-(*S*)
Empirical formula	$C_{27}H_{33}NO$	$C_{27}H_{33}NO$	$C_{27}H_{33}NO$
Formula weight	387.54	387.54	387.54
Crystal system	Monoclinic	Monoclinic	Monoclinic
Space group	$P2_1/n$	$P2_1$	$P2_1$
a (Å)	6.26540(10)	6.2610(6)	6.2495(3)
b (Å)	9.92340(10)	9.8995(8)	9.9089(4)
c (Å)	38.5420(4)	19.794(2)	19.7638(9)
α (°)	90	90	90
β (°)	94.1590(10)	96.340(9)	96.376(4)
γ (°)	90	90	90
Volume (Å^3)	2390.00(5)	1219.3(2)	1216.32(9)
Z	4	2	2
GOF	1.003	1.002	1.004
R_1 [$I > 2\sigma(I)$]	0.0536	0.0800	0.0625
wR_2 [$I > 2\sigma(I)$]	0.1469	0.2435	0.1708

Table S4. Pictures of photochromic behavior for SA-NPh-(*R*), SA-NPh-(*S*), and SA-NPh-(*Rac*). These compounds do not show significant color changes as they transform from Phase I to Phase II. However, under UV irradiation of 365 nm, they can change from yellow (Phase I) to orange (Phase III).

Compound	Temperature		Optical radiation	
	Low temperature	High temperature	vis	UV (365 nm)
SA-NPh-(*R*)	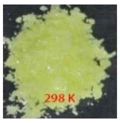	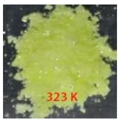	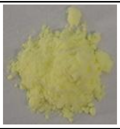	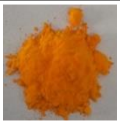
SA-NPh-(*S*)	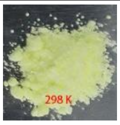	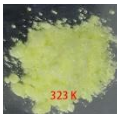	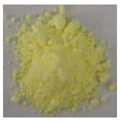	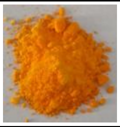
SA-NPh-(*Rac*)	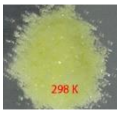	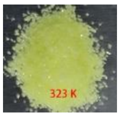	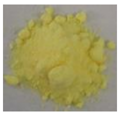	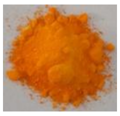
