# Supplementary material for: Domain memory effect in the organic ferroics
Source: Nat Commun. 2022 May 2;13:2379. doi: 10.1038/s41467-022-30085-1 (PMC9061795; doi:10.1038/s41467-022-30085-1)
Supplement: Supplementary file 3 — Reporting Summary [file 41467_2022_30085_MOESM3_ESM.pdf]

nature portfolio

Corresponding author(s): Ren-Gen Xiong; Zhong-Xia Wang

Last updated by author(s): Apr 15, 2022

Reporting Summary

Nature Portfolio wishes to improve the reproducibility of the work that we publish. This form provides structure for consistency and transparency in reporting. For further information on Nature Portfolio policies, see our Editorial Policies and the Editorial Policy Checklist.

Statistics

For all statistical analyses, confirm that the following items are present in the figure legend, table legend, main text, or Methods section.

n/a	Confirmed	
☐	☒	The exact sample size (*n*) for each experimental group/condition, given as a discrete number and unit of measurement
☐	☒	A statement on whether measurements were taken from distinct samples or whether the same sample was measured repeatedly
☐	☒	The statistical test(s) used AND whether they are one- or two-sided<br>*Only common tests should be described solely by name; describe more complex techniques in the Methods section.*
☒	☐	A description of all covariates tested
☒	☐	A description of any assumptions or corrections, such as tests of normality and adjustment for multiple comparisons
☐	☒	A full description of the statistical parameters including central tendency (e.g. means) or other basic estimates (e.g. regression coefficient) AND variation (e.g. standard deviation) or associated estimates of uncertainty (e.g. confidence intervals)
☒	☐	For null hypothesis testing, the test statistic (e.g. *F*, *t*, *r*) with confidence intervals, effect sizes, degrees of freedom and *P* value noted<br>*Give P values as exact values whenever suitable.*
☒	☐	For Bayesian analysis, information on the choice of priors and Markov chain Monte Carlo settings
☒	☐	For hierarchical and complex designs, identification of the appropriate level for tests and full reporting of outcomes
☒	☐	Estimates of effect sizes (e.g. Cohen's *d*, Pearson's *r*), indicating how they were calculated

Our web collection on statistics for biologists contains articles on many of the points above.

Software and code

Policy information about availability of computer code

Data collection	No software was used
Data analysis	No software was used

For manuscripts utilizing custom algorithms or software that are central to the research but not yet described in published literature, software must be made available to editors and reviewers. We strongly encourage code deposition in a community repository (e.g. GitHub). See the Nature Portfolio guidelines for submitting code & software for further information.

Data

Policy information about availability of data

All manuscripts must include a data availability statement. This statement should provide the following information, where applicable:

- Accession codes, unique identifiers, or web links for publicly available datasets
- A description of any restrictions on data availability
- For clinical datasets or third party data, please ensure that the statement adheres to our policy

All data generated and analyzed in this study are included in the Article and its Supplementary Information, and are also available from corresponding authors upon request. The crystal structures generated in this study have been deposited in the Cambridge Crystallographic Data Centre under accession code CCDC: 2070777-2070781, 2070783-2070785, and can be obtained free of charge from the CCDC via www.ccdc.cam.ac.uk/data_request/cif.

Field-specific reporting

Please select the one below that is the best fit for your research. If you are not sure, read the appropriate sections before making your selection.

☐ Life sciences ☐ Behavioural & social sciences ☒ Ecological, evolutionary & environmental sciences

For a reference copy of the document with all sections, see nature.com/documents/nr-reporting-summary-flat.pdf

Ecological, evolutionary & environmental sciences study design

All studies must disclose on these points even when the disclosure is negative.

Study description	We present a pair of single-component organic enantiomorphic ferroelectric/ferroelastic crystals, demonstrating the existence of memory effect for their ferroic domains.
Research sample	(R)- and (S)-N-3,5-di-tert-butylsalicylidene-1-(1-naphthyl)ethylamine (SA-NPh-(R) and SA-NPh-(S))
Sampling strategy	The single crystals of SA-NPh-(R) and SA-NPh-(S) used in the article were obtained by slowly evaporating the methanol solution at room temperature.
Data collection	Z.-X.W. synthesized the samples. X.-J.S. and Y.-Y.T. carried out the PFM study. X.-G.C. and Y.-L.Z. carried out the general characterizations. P.-F.L. and W.-Q.L. carried out the theoretical analysis. R.-G.X. conceived the study.
Timing and spatial scale	Data collection for this article began in September 2020 and ended in March 2021. The crystal sample was firstly synthesized, and then its physical properties were characterized, including phase transition, ferroelectric and optical properties. Each characterization experiment process involves many repetitions, so this step takes the longest time, which is complex and has no specific sequence.
Data exclusions	No data were excluded from the analyses.
Reproducibility	All attempts to repeat the experiment were successful.
Randomization	The randomization is not relevant to our study, because the structure of crystal samples obtained by repeated synthesis is determined, and the purity can reach 100%.
Blinding	We have arranged for two authors to conduct experiments independently, characterizing samples and collecting data at different times, to ensure that the final results are consistent.

Did the study involve field work? ☐ Yes ☒ No

Reporting for specific materials, systems and methods

We require information from authors about some types of materials, experimental systems and methods used in many studies. Here, indicate whether each material, system or method listed is relevant to your study. If you are not sure if a list item applies to your research, read the appropriate section before selecting a response.

Materials & experimental systems

n/a	Involved in the study
☒	☐ Antibodies
☒	☐ Eukaryotic cell lines
☒	☐ Palaeontology and archaeology
☒	☐ Animals and other organisms
☒	☐ Human research participants
☒	☐ Clinical data
☒	☐ Dual use research of concern

Methods

n/a	Involved in the study
☒	☐ ChIP-seq
☒	☐ Flow cytometry
☒	☐ MRI-based neuroimaging
